# Supplementary material for: All-organic polymeric materials with high refractive index and excellent transparency
Source: Nat Commun. 2023 Jun 14;14:3524. doi: 10.1038/s41467-023-39125-w (PMC10267154; doi:10.1038/s41467-023-39125-w)
Supplement: Supplementary file 1 — Supplementary Information [file 41467_2023_39125_MOESM1_ESM.pdf]

Supplementary Information

All-Organic Polymeric Materials with High Refractive Index and Excellent Transparency

Jie Zhang[1‡], Tianwen Bai[1‡], Weixi Liu[2‡], Mingzhao Li[3,4], Qiguang Zang[1], Canbin Ye[3,4], Jing Zhi Sun[1], Yaocheng Shi[2], Jun Ling[1], Anjun Qin[3,4]*, and Ben Zhong Tang[1,3,4,5]*

[1] MOE Key Laboratory of Macromolecules Synthesis and Functionalization, Department of Polymer Science and Engineering, Zhejiang University, Hangzhou 310027, China

[2] College of Optical Science and Engineering and International Research Center for Advanced Photonics, Zhejiang University, Hangzhou 310058, China.

[3] State Key Laboratory of Luminescent Materials and Devices, Guangdong Provincial Key Laboratory of Luminescence from Molecular Aggregates, South China University of Technology, Guangzhou 510640, China. E-mail: msqinaj@scut.edu.cn

[4] Center for Aggregation-Induced Emission, South China University of Technology, Guangzhou 510640, China.

[5] School of Science and Engineering, Shenzhen Institute of Aggregate Science and Technology, The Chinese University of Hong Kong, Shenzhen, Guangdong 518172, China. E-mail: tangbenz@cuhk.edu.cn

‡ These authors contributed equally.

Contents

1. **Supplementary experimental details** S3
2. **Supplementary figures** S9

Supplementary Fig. 1. Synthetic routes to monomers 1. S9

Supplementary Fig. 2. TGA and DSC thermograms of polymers P1-P5. S9

Supplementary Fig. 3. FT-IR spectra of monomer 1b, 2a, and P2. S9

Supplementary Fig. 4. Synthetic route to model compound 4. S10

Supplementary Fig. 5. ^{1}H and ^{13}C NMR spectra of 1b, 2a, 4 and P2. S10

Supplementary Fig. 6. FT-IR spectra of monomer of P1, P3-P5. S10

Supplementary Fig. 7. ^{1}H NMR spectrum of P1, P3-P5 in $CDCl_3$. S11

Supplementary Fig. 8. ^{13}C NMR spectrum of P1, P3-P5 in $CDCl_3$. S12

Supplementary Fig. 9. Synthetic route to the intermediate 5. S12

Supplementary Fig. 10. ^{1}H and ^{13}C NMR spectra of compound 5 in$CDCl_3$. S13

Supplementary Fig. 11. Mass spectrum of intermediate 5. S13

Supplementary Fig. 12. Synthetic route to model compound 6. S13

Supplementary Fig. 13. ^{1}H and ^{13}C NMR spectra of compound 6 in$CDCl_3$. S14

Supplementary Fig. 14. HPLC spectra of the model crude product 6. S14

Supplementary Fig. 15. SEM picture for cross section of P3 film on glass. S14

Supplementary Fig. 16. Pictures of intermediates and transition states. S15

3. **Supplementary tables** S16

Supplementary Table 1. Temperature dependence on the polymerization. S16

Supplementary Table 2. Effect of monomers concentration on the polymerization. S16

Supplementary Table 3. Time dependence on the polymerization. S17

Supplementary Table 4. Solvent dependence on the polymerization. S17

Supplementary Table 5. Crystal data and structure refinement for single crystals. S18

Supplementary Table 6. Measured propagation losses of P3, P4 and SU-8 waveguides. S19

4. **Optimized geometries by B3LYP/6-311++G(d,p) in reaction route.** S20

1 Supplementary experimental details

Synthesis and characterization of monomers. The synthetic procedures of 1b-1d are resemble with that of 1a (Supplementary Fig. 1). The detailed procedure for the synthesis of 1a was given here as an example.

1-Chloro-4-ethynylbenzene (7, 1.637 g, 12.0 mmol) was dissolved in acetone (50 mL), and *N*-bromosuccinimide (3.204 g, 18.0 mmol) and $AgNO_3$ (204 mg, 1.2 mmol) were added. The reaction mixture was stirred overnight at room temperature in dark. Afterwards, the solution was concentrated under reduced pressure and the crude product was purified by silica gel column chromatography using petroleum ether (PE) as eluent. White powder of 1a was obtained.

Characterization data of 1a: 1a was obtained as white powder in 81.3% yield (2.100 g). IR (KBr), ν (cm^{-1}): 2195 (C≡C stretching). ^1H NMR (500 MHz, $CDCl_3$) δ (TMS, ppm): 7.37 (d, J = 8.7 Hz, 2H), 7.28 (d, J = 8.7 Hz, 2H). ^{13}C NMR (125 MHz, $CDCl_3$) δ (ppm):134.80, 133.21, 128.71, 121.18, 78.98, 51.02.

Characterization data of 1b: 1b was obtained as light yellow powder in 84.8% yield (2.684 g). IR (KBr), ν (cm^{-1}): 2048 (C≡C stretching). ^1H NMR (500 MHz, $CDCl_3$) δ (TMS, ppm): 7.38 (d, J = 8.9 Hz, 2H), 6.83 (d, J = 8.7 Hz, 2H), 3.81(s, 3H). ^{13}C NMR (125 MHz, $CDCl_3$) δ (ppm):160.11, 133.66, 114.69,113.95, 79.94, 55.35, 47.79.

Characterization data of 1c: 1c was obtained as white powder in 91.6% yield (2.348 g). IR (KBr), ν (cm^{-1}): 2197 (C≡C stretching). ^1H NMR (500 MHz, $CDCl_3$) δ (TMS, ppm): 7.44 (d, J = 8.5 Hz, 2H), 7.29 (d, J = 8.5 Hz, 2H). ^{13}C NMR (125 MHz, $CDCl_3$) δ (ppm):133.38, 131.65, 123.05, 121.66, 79.08, 51.26.

Characterization data of 1d: 1d was obtained as yellow powder in 73.1% yield (317.2 mg). IR (KBr), v (cm^{-1}): 2191 (C≡C stretching). ^1H NMR (400 MHz, $CDCl_3$) δ (TMS, ppm): 7.17 (d, *J* = 8.4 Hz, 2H), 7.10 (m, 9H), 7.01 (m, 6H), 6.96 (d, *J* = 8.4 Hz, 2H). ^{13}C NMR (125 MHz, $CDCl_3$) δ (ppm):144.42, 143.42, 143.23, 141.81, 140.11, 131.35-131.28, 127.84, 127.78, 127.69, 126.73, 126.65, 126.62, 120.47, 80.21, 49.74.

Synthesis and characterization of model compound 4. To facilitate the structural characterization, model compound 4 was prepared under the same reaction conditions (Supplementary Fig. 4). Compound 1b (63.3 mg, 0.3 mmol) and 11 (82.0 mg, 0.66 mmol) were placed into a 20 mL Schlenk tube. DMSO (3.0 mL) was injected into the tube to dissolve the monomers, and then DBU (157 μL, 1.05 mmol) was injected. Afterwards, the mixture was stirred at 80 °C for 4 h under nitrogen. After cooling down to room temperature, the reaction mixture was extracted with DCM (25 mL, three times) and the combined organic layer was washed with water, and dried over $MgSO_4$. After filtration and solvent evaporation, the crude product was purified by silica gel column chromatography using PE as eluent. Finally, 4 was obtained as white powder in 71.2% yield (80.7 mg). ^1H NMR (500 MHz, $CDCl_3$) δ 7.46 (d, *J* = 8.8 Hz, 2H), 7.39 (d, *J* = 8.1 Hz, 2H), 7.16 (d, *J* = 8.1 Hz, 2H), 7.14 (d, *J* = 8.2 Hz, 2H), 7.02 (s, 1H), 6.99 (d, *J* = 8.0 Hz, 2H), 6.76 (d, *J* = 8.8 Hz, 2H), 3.75 (s, 3H), 2.35 (s, 3H), 2.24 (s, 3H). ^{13}C NMR (500 MHz, $CDCl_3$) δ (ppm): 159.11, 137.55, 135.73, 134.38, 131.97, 131.59, 131.21, 130.81, 129.98, 129.64, 129.19, 128.58, 128.02, 113.80, 55.21, 22.11, 20.99.

Synthesis and characterization of intermediate 5. To explore the reaction mechanism, we attempted to catch intermediate of the reaction. The intermediate 5 was finally obtained under the following reaction conditions (Supplementary Fig. 9). Compound 1c (78.0 mg, 0.3 mmol) and 11 (37.2 mg, 0.3 mmol) were placed into a 20 mL Schlenk tube. After being evacuated and refilled with nitrogen for three times, DMSO (3.0 mL) was injected into the tube to dissolve the monomers, and then DBU (45 μL, 0.3 mmol) was injected. Afterwards, the mixture was stirred at 80 °C for 0.5 h. After cooling down to room temperature, the reaction mixture was extracted with DCM (25 mL, three times) and the combined organic layer was washed with water, and dried over $MgSO_4$. After filtration and solvent evaporation, the crude product was purified by silica gel column chromatography using PE as eluent. Finally, 5 was obtained as white powder in 12.7% yield (11.5 mg). ^1H NMR (500 MHz, $CDCl_3$) δ (TMS, ppm): 7.47 (d, J = 8.6 Hz, 2H), 7.37 (d, J = 8.1 Hz, 2H), 7.34 (d, J = 8.6 Hz, 2H), 7.17 (d, J = 8.1 Hz, 2H), 2.35 (s, 3H). ^{13}C NMR (125 MHz, $CDCl_3$) δ (TMS, ppm): 136.88, 132.98, 131.64, 130.11, 128.74, 126.79, 122.72, 121.98, 95.99, 77.82, 21.02. TOF MS (m/z): [M^+] calcd. for $C_{15}H_{11}SBr$, 301.9765; found, 301.9767.

Synthesis and characterization of model compound 6. To define the reaction, model compound 6 was prepared under the same reaction conditions as polymerization (Supplementary Fig. 12). 1c (78.0 mg, 0.3 mmol) and 11 (82.0 mg, 0.66 mmol) were placed into a 20 mL Schlenk tube. After being evacuated and refilled with nitrogen for three times, DMSO (3.0 mL) was injected into the tube to dissolve the monomers, and then DBU (157 μL, 1.05 mmol) was injected. Afterwards,

the mixture was stirred at 80 °C for 4 h. After cooling to room temperature, the reaction mixture was extracted with DCM (25 mL, three times) and the combined organic layer was washed with water, and dried over $MgSO_4$. The solvent was evaporated and the crude product was purified by silica gel column chromatography using PE as eluent. 6 was obtained as white powder in 83.5% yield (107.0 mg). The crystal data and structure refinement for single crystals of compound 6 was shown in Supplementary Table 5. ^{1}H NMR (500 MHz, $CDCl_3$) δ (TMS, ppm): 7.40 (d, J = 8.0 Hz, 2H), 7.39 (d, J = 8.5 Hz, 2H), 7.34 (d, J = 8.5 Hz, 2H), 7.18 (d, J = 8.0 Hz, 2H), 7.14 (s, 1H), 7.12 (d, J = 8.5 Hz, 2H), 7.00 (d, J = 8.0 Hz, 2H), 2.36 (s, 3H), 2.25 (s, 3H). ^{13}C NMR (125 MHz, $CDCl_3$) δ (TMS, ppm): 137.98, 137.80, 137.61, 136.13, 131.42, 131.12, 130.59, 130.08, 129.75, 128.72, 128.27, 128.02, 121.32, 21.12, 20.99.

Polymer Synthesis. All the polymerization reactions were carried out under nitrogen using a standard Schlenk technique. A typical procedure for the polymerization of **1a** and **2a** is given below as an example.

The monomers of 1a (43.1 mg, 0.2 mmol) and 2a (50.1 mg, 0.2 mmol) were placed into a 10 mL Schlenk tube. After being evacuated and refilled with nitrogen for three times, DMSO (1.0 mL) was injected into the tube to dissolve the monomers, and then DBU (30 μL, 0.2 mmol) was injected. The mixture was stirred at 80 °C for 4 h. After cooled down to room temperature, the resultant solution was diluted with THF (5 mL), and then added dropwise into 200 mL methanol through a cotton filter under stirring. The precipitate was allowed to stand overnight and then collected by filtration. The polymer was washed with methanol and dried to a constant weight, affording a white

powder product. The detailed characterization data were given in the supporting information.

Characterization of polymers. Characterization Data of P1: white powder (75.6 mg); yield 98.5% (Table 1, no. 1); M_w: 44500. M_w/M_n: 1.87. FT-IR (KBr), v (cm^{-1}): 3061, 3010, 1894, 1638, 1566, 1534, 1473, 1389, 1296, 1178, 1092, 1006, 924, 810, 743, 706, 541. ^1H NMR (500 MHz, $CDCl_3$) δ (TMS, ppm): 7.40, 7.30, 7.15, 7.08, 7.06, 7.03. ^{13}C NMR (125 MHz, $CDCl_3$) δ (ppm): 136.81, 136.40, 136.16, 135.34-132.20, 131.82, 131.43, 131.21, 130.74, 129.16, 128.69, 128.02.

Characterization Data of P2: white powder (72.5 mg); yield 95.4% (Table 3, no. 2); M_w: 11200. M_w/M_n: 1.59. FT-IR (KBr), v (cm^{-1}): 3041, 3010, 1893, 1571, 1535, 1473, 1388, 1297, 1180, 1090, 1011, 926, 809, 741, 708, 541. ^1H NMR (500 MHz, $CDCl_3$) δ (TMS, ppm): 7.43, 7.38,7.37, 7.34-7.27, 7.15, 7.11, 7.10, 7.04, 6.76, 3.74. ^{13}C NMR (125 MHz, $CDCl_3$) δ (ppm): 159.43, 136.01-131.34, 130.81, 129.46, 129.00, 128.49, 128.09, 113.80, 55.27.

Characterization Data of P3: white powder (84.4 mg); yield 98.1% (Table 3, no. 3); M_w: 38600. M_w/M_n: 1.98. FT-IR (KBr), v (cm^{-1}): 3058, 3007,1895,1636,1567, 1532, 1471, 1384, 1261, 1178, 1095, 1069, 1004, 922, 805, 741, 698, 541. ^1H NMR (500 MHz, $CDCl_3$) δ (TMS, ppm): 7.34, 7.25, 7.16, 7.15, 7.11-7.03. ^{13}C NMR (125 MHz, $CDCl_3$) δ (ppm): 137.24, 136.88-132.22, 131.77, 131.60, 131.42, 131.20, 130.75, 129.14, 128.27, 127.05, 121.85.

Characterization Data of P4: yellow powder (116.9 mg); yield 96.6% (Table 3, no. 4); M_w: 11500. M_w/M_n: 1.60. FT-IR (KBr), v (cm^{-1}): 3055, 3024, 2164, 1889, 1567,

1468,1440, 1388, 1330, 1178, 1146, 1090, 1069, 1006, 918, 810, 752, 696, 617, 574, 543. ^{1}H NMR (500 MHz, $CDCl_3$) δ (TMS, ppm): 7.35, 7.22, 7.08, 7.05, 7.00-6.94, 6.88, 3.49. ^{13}C NMR (125 MHz, $CDCl_3$) δ (ppm): 144.61, 143.52, 143.41, 141.93, 141.36, 140.18, 136.07-131.71, 131.48, 131.30, 131.13, 131.02, 130.79, 130.68, 129.14, 128.48, 127.89, 127.73, 126.93, 126.53, 126.12, 120.37.

Characterization Data of P5: white powder (79.0 mg); yield 99.2% (Table 3, no. 5); M_w: 19800. M_w/M_n: 2.07. FT-IR (KBr), ν (cm^{-1}): 3066, 3021, 2592, 2164, 1896, 1591, 1531, 1474, 1387, 1082, 1002, 921, 804, 631, 572, 541. ^{1}H NMR (500 MHz, $CDCl_3$) δ (TMS, ppm): 7.57, 7.56, 7.55, 7.54, 7.52, 7.50, 7.48-7.38, 7.37, 7.31, 7.27. ^{13}C NMR (125 MHz, $CDCl_3$) δ (ppm): 137.50, 133.11, 131.61, 131.05, 128.56, 128.18, 127.77-127.31, 126.81, 122.97, 121.68, 96.86.

2 Supplementary figures

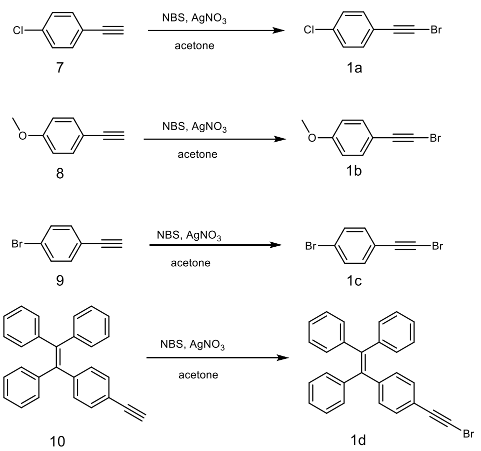

Supplementary Fig. 1. Synthetic routes to monomers 1.

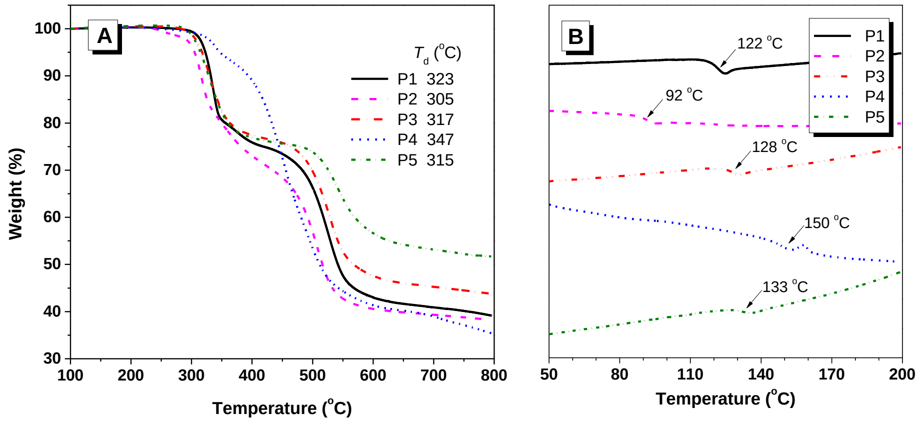

Supplementary Fig. 2. (A) TGA and (B) DSC curves of P1-P5. T_d represents the temperatures of 5% weight loss. T_g represents glass-transition temperatures.

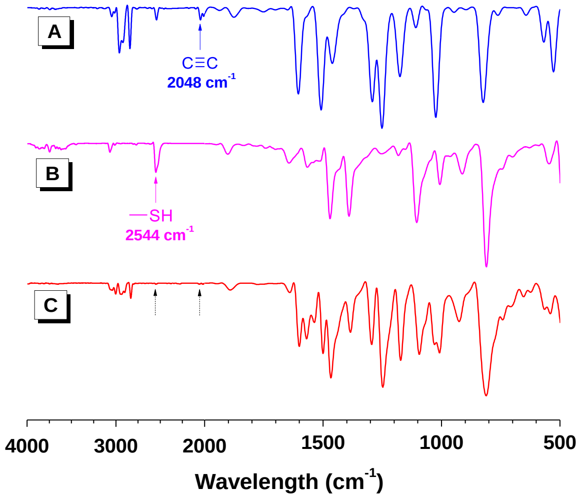

Supplementary Fig. 3. FT-IR spectra of monomers 1b (A) and 2a (B), and P2 (C).

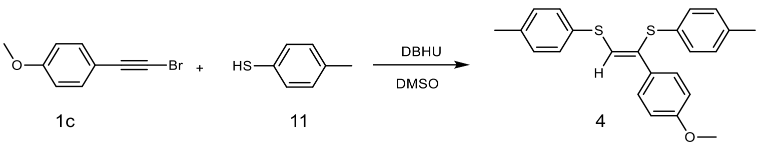

Supplementary Fig. 4. Synthetic route to model compound 4.

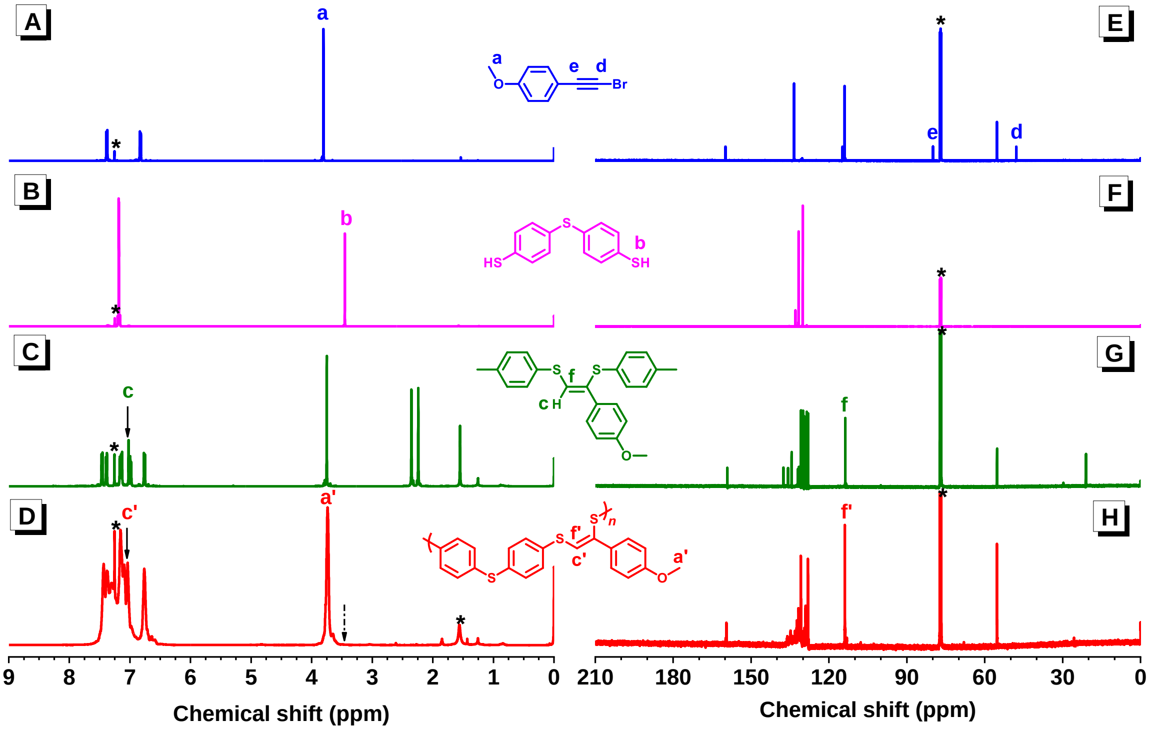

Supplementary Fig. 5. ^{1}H NMR and ^{13}C NMR spectra of monomer 1b (A, E), monomer 2a (B, F), model compound 4 (C, G) and P2 (D, H), respectively. The solvent peaks are marked with asterisks.

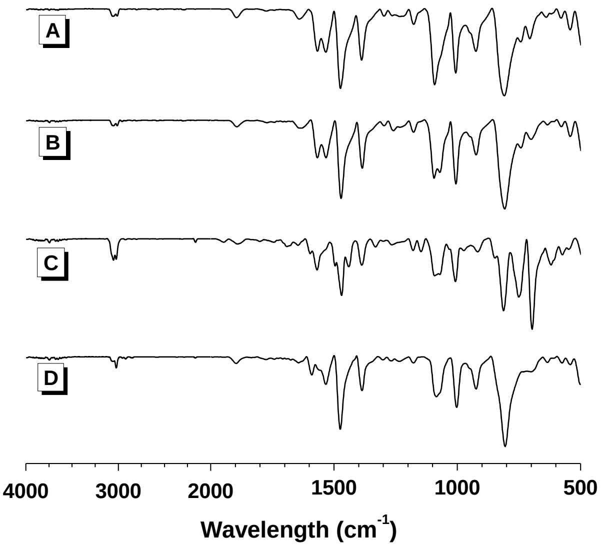

Supplementary Fig. 6. FT-IR spectra of P1 (A), P3 (B), P4 (C) and P5 (D).

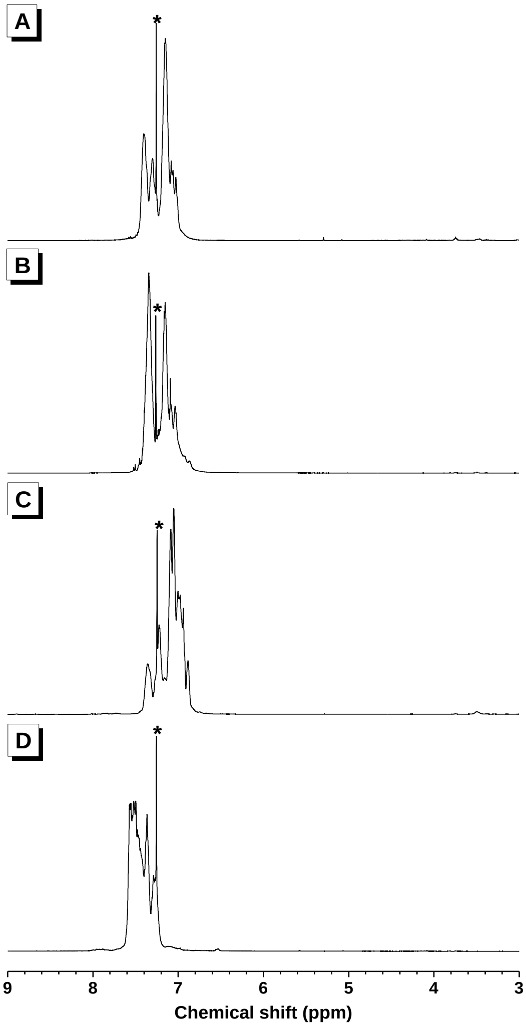

Supplementary Fig. 7. ^{1}H NMR spectra of P1 (A), P3 (B), P4 (C) and P5 (D) in $CDCl_3$. The solvent peaks are marked with asterisks.

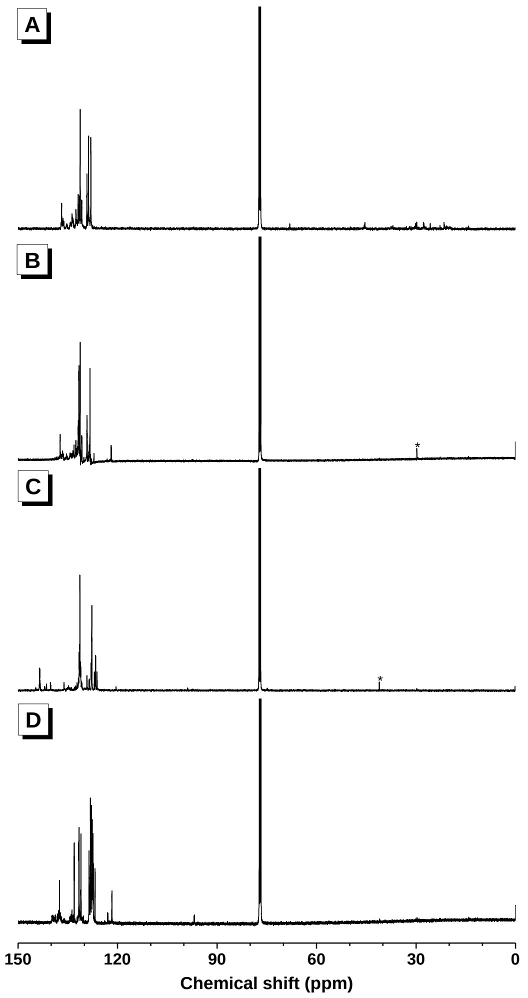

Supplementary Fig. 8. ^{13}C NMR spectra of P1 (A), P3 (B), P4 (C) and P5 (D) in $CDCl_3$. The solvent peaks are marked with asterisks.

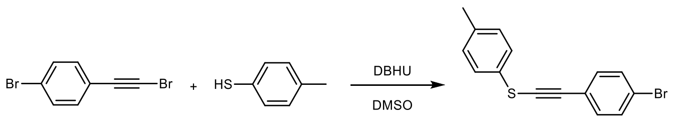

Supplementary Fig. 9. Synthetic route to the intermediate 5.

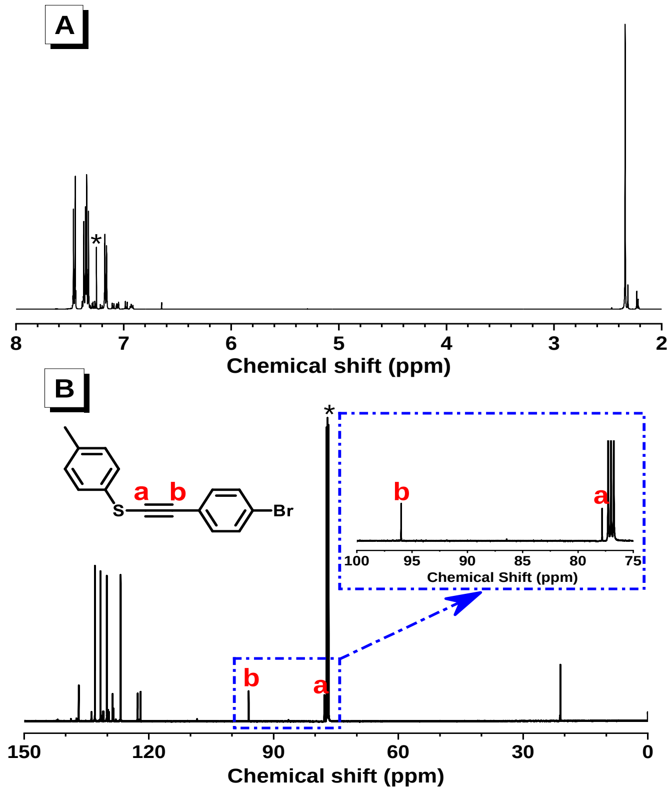

Supplementary Fig. 10. ^{1}H (A) and ^{13}C NMR (B) spectra of intermediate 5 in $CDCl_3$. The solvent peaks are marked with asterisks

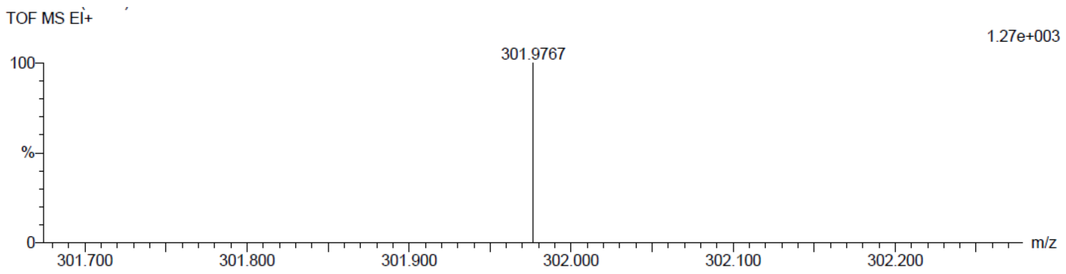

Minimum:				-1.5		
Maximum:		1.0	10.0	50.0		
Mass	Calc. Mass	mDa	PPM	DBE	i-FIT	Formula
301.9767	301.9765	0.2	0.7	10.0	5546660.0	C15 H11 S Br

Supplementary Fig. 11. Mass spectrum of intermediate 5.

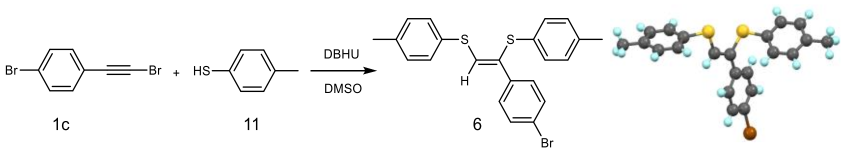

Supplementary Fig. 12. Synthetic route to model compound 6.

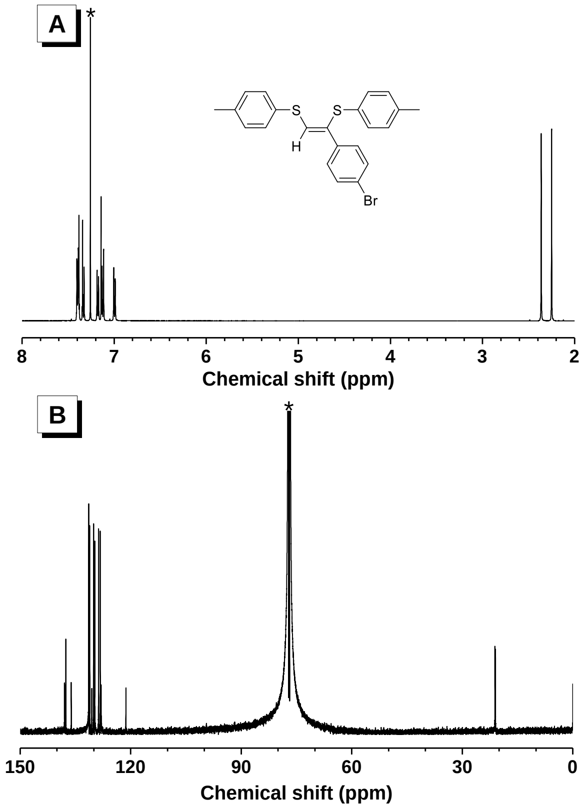

Supplementary Fig. 13. ^{1}H NMR (A) and ^{13}C NMR (B) spectra of model compound 6 in $CDCl_3$. The solvent peaks are marked with asterisks.

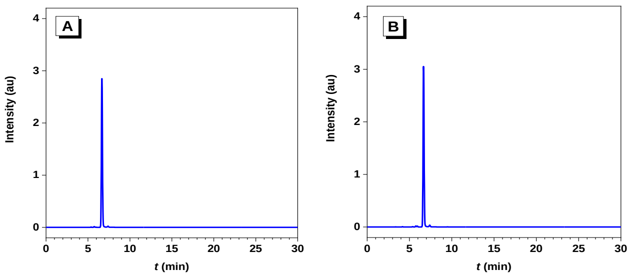

Supplementary Fig. 14. HPLC spectra of the model crude product **6** with detector of 320 nm UV light (A) and 254 nm UV light (B).

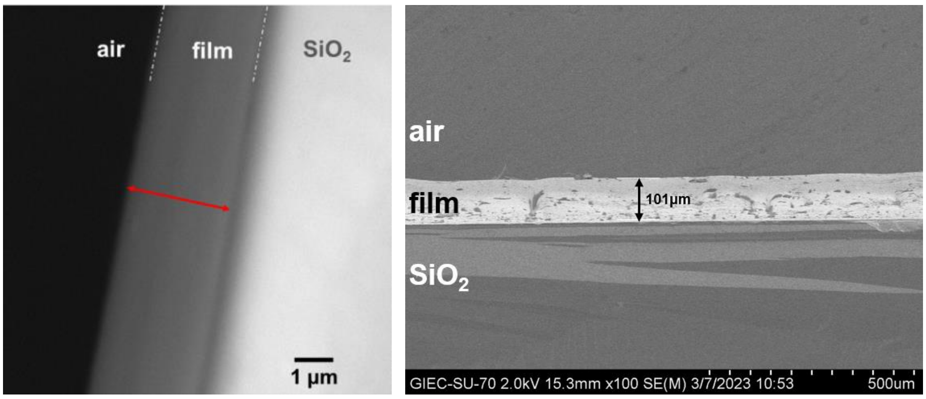

Supplementary Fig. 15. SEM pictures for cross sections of P3 films on glass.

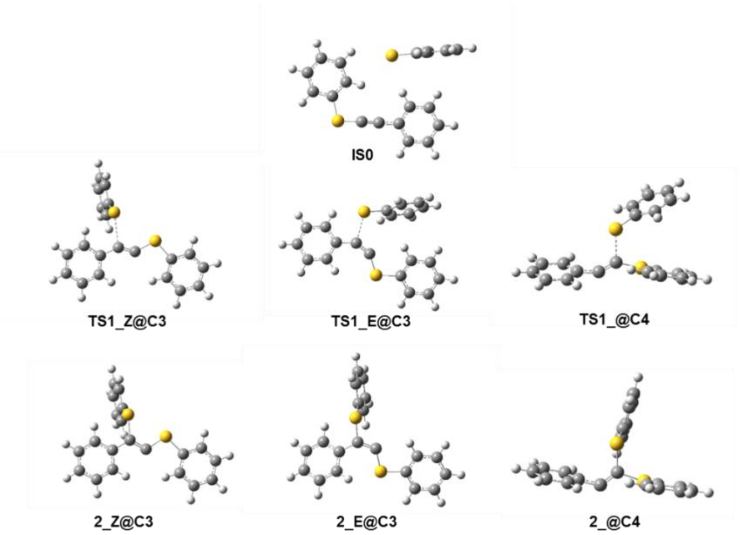

Supplementary Fig. 16. The structures of intermediates and transition states.

3 Supplementary Tables

Supplementary Table 1. The effect of reaction temperature on the polymerization results [a]

entry	T (°C)	yield (%)	M_w [b]	$Đ$ [b]
1	60	trace	-	-
2	70	63.2	8900	2.08
3	80	96.4	9600	2.34
4	90	77.4	15400	3.81

[a] Carried out in DMSO under nitrogen for 3 h ([1a]/[2a] = 1:1, [1a] = 0.1 M, [DBU]/[1a] = 1). [b] M_w and $Đ$ (M_w/M_n) of polymers were estimated by GPC in THF on the basic of a polymethyl methacrylate calibration.

Supplementary Table 2. Effect of monomer concentration on the polymerization results [a]

entry	[M] (M)	yield (%)	M_w [b]	$Đ$ [b]
1	0.1	90.6	13200	3.25
2	0.2	88.8	15500	2.08
3	0.3	94.0	5400	4.04

[a] Carried out in DMSO under nitrogen at 80 °C for 3 h ([1a]/[2a] = 1:1, [DBU]/[1a] = 1. [b] M_w and $Đ$ (M_w/M_n) of polymers were estimated by GPC in THF on the basic of a polymethyl methacrylate calibration.

Supplementary Table 3. The effect of reaction time on the polymerization results [a]

entry	*t* (h)	yield (%)	M_w [b]	*Đ* [b]
1	3	90.8	14100	3.50
2	4	94.9	37000	1.77
3	5	90.0	18100	2.71
4	6	90.9	15000	2.59

[a] Carried out in DMSO at 80 °C under nitrogen ([1a]/[2a] = 1:1, [1a] = 0.2 M, [DBU]/[1a] = 1. [b] M_w and *Đ* (M_w/M_n) of polymers were estimated by GPC in THF on the basic of a polymethyl methacrylate calibration.

Supplementary Table 4. Solvent dependence of the polymerization [a]

entry	solvent	M_w [b]	*Đ* [b]	yield (%)
1	DMSO	44500	1.87	98.5
2	DMF	42400	2.44	97.1
3	NMP	9400	1.84	94.9

[a] Carried out under nitrogen at 80 °C for 4 h ([1a]/[2a] = 1:1, [1a] = 0.2, [DBU]/[1a] = 1. [b] M_w and *Đ* (M_w/M_n) of polymers were estimated by GPC in THF on the basic of a polymethyl methacrylate calibration.

Supplementary Table 5. Crystal data and structure refinement for single crystals

Crystal form	Model compound 6
CCDC number	1889353
Empirical formula	$C_{22}H_{19}BrS_2$
Formula weight	427.42
Temperature (K)	149.99(10)
Wavelength (Å)	1.54184
Crystal system, space group	Monoclinic, $P2_1/n$
Unit cell dimensions	a = 13.4017(2) Å<br>b = 5.92106(11) Å<br>c = 24.3917(4) Å<br>α = 90°<br>β = 90.1424(14)°<br>γ = 90°
Volume (Å^3)	1935.53(5)
Z, Calculated density (g cm^{-3})	4, 1.467
Absorption coefficient (mm^{-1})	4.904
F(000)	872.0
2Θ range for data collection/°	7.248 to 134.106
Index ranges	$-15 \leq h \leq 16$, $-4 \leq k \leq 7$, $-29 \leq l \leq 28$
Reflections collected	11318
Independent reflections	3435 [R_{int} = 0.0221, R_{sigma} = 0.0204]
Data/restraints/parameters	3435/0/228
Goodness-of-fit on F^2	1.074
Final R indexes [I>=2σ (I)]	R_1 = 0.0342, wR_2 = 0.0887
Final R indexes [all data]	R_1 = 0.0357, wR_2 = 0.0897

Supplementary Table 6. Measured propagation losses of P3, P4 and SU-8 waveguides @ 1550 nm.

P3		P4		SU-8	
w (mm)	L_{prop} (dB)	w (mm)	L_{prop} (dB)	w (mm)	L_{prop} (dB)
0.095	0	0.4	-0.2	0.778	-1.67
2.020	-1.40	1.7	-1.1	3.137	-4.72
4.902	-3.50	2.4	-1.6	5.508	-8.5
7.782	-4.70	5.0	-4.4	11.004	-14.9
11.724	-7.70	8.7	-7.5		
		11.7	-11.2		

4 Optimized geometries by B3LYP/6-311++G(d,p) in reaction route.

IS0	C	-2.214831	2.048091	0.000075
	C	-1.036220	2.334838	0.000006
	S	0.742484	-2.163091	-0.001760
	C	2.489834	-2.023166	-0.000520
	C	3.238833	-1.955049	1.201696
	C	3.240452	-1.954409	-1.201691
	C	4.623482	-1.817010	1.201137
	H	2.699131	-2.008102	2.141022
	C	4.625097	-1.816372	-1.199205
	H	2.702010	-2.006968	-2.141767
	C	5.339131	-1.742584	0.001467
	H	5.152689	-1.764921	2.149673
	H	5.155576	-1.763787	-2.147003
	H	6.418932	-1.634330	0.002223
	C	0.352583	2.650029	-0.000050
	C	1.311707	1.619353	-0.000201
	C	0.779988	3.991282	0.000057
	C	2.667195	1.935822	-0.000232
	H	0.996965	0.577831	-0.000333
	C	2.137681	4.292403	0.000014
	H	0.041644	4.785237	0.000170
	C	3.085204	3.267134	-0.000126
	H	3.391015	1.128676	-0.000343
	H	2.456319	5.329879	0.000093
	H	4.143861	3.504541	-0.000153
	S	-3.860917	1.693857	0.000181
	C	-3.923219	-0.110542	0.000172
	C	-5.204304	-0.673653	0.000446
	C	-2.790610	-0.920953	-0.000098
	C	-5.343559	-2.058688	0.000442
	H	-6.083455	-0.036959	0.000666
	C	-2.943541	-2.307890	-0.000095
	H	-1.791547	-0.503053	-0.000304
	C	-4.214326	-2.879724	0.000170
	H	-6.337474	-2.494402	0.000659
	H	-2.048843	-2.920862	-0.000296
	H	-4.325362	-3.958497	0.000169

TS1_Z@C3

C	1.019957	0.416348	-0.357193
C	-0.144691	0.884468	-0.479765
S	-1.715915	-0.821570	-1.642220
C	-2.648895	-1.596592	-0.362505
C	-2.450303	-1.315581	1.007079
C	-3.641856	-2.552571	-0.677573
C	-3.200462	-1.948841	1.994471
H	-1.689890	-0.596123	1.286357
C	-4.385389	-3.185744	0.313253
H	-3.817735	-2.789617	-1.721357
C	-4.176365	-2.891154	1.663328
H	-3.016446	-1.703816	3.037056
H	-5.138520	-3.915761	0.028049
H	-4.758136	-3.383373	2.435789
C	-0.927793	2.077383	-0.236530
C	-2.275779	2.238180	-0.595895
C	-0.289145	3.161585	0.410433
C	-2.951279	3.425810	-0.323698
H	-2.767960	1.412950	-1.094668
C	-0.969359	4.342230	0.677917
H	0.749624	3.050046	0.696243
C	-2.310249	4.487065	0.312510
H	-3.993550	3.517207	-0.613254
H	-0.449883	5.155065	1.176699
H	-2.842522	5.408910	0.523497
S	1.847722	-1.026325	-0.678220
C	3.525682	-0.707823	-0.137242
C	4.442402	-1.762096	-0.257358
C	3.958786	0.515958	0.382163
C	5.766340	-1.592066	0.135900
H	4.113180	-2.715849	-0.658034
C	5.285829	0.677979	0.773652
H	3.241486	1.323635	0.470367
C	6.199180	-0.370138	0.654722
H	6.462808	-2.418798	0.037498
H	5.607809	1.633889	1.175344
H	7.231158	-0.238439	0.961438

TS1_E@C3

C	0.429819	-0.772554	-0.003956
C	1.538923	-0.181720	-0.043157
S	1.431254	2.204592	-0.520860
C	-0.242176	2.539290	-0.117300
C	-0.787771	2.189877	1.135950
C	-1.097465	3.185316	-1.035135
C	-2.103705	2.505760	1.464131
H	-0.157424	1.673351	1.848914
C	-2.409383	3.509063	-0.699822
H	-0.707138	3.437033	-2.015045
C	-2.925768	3.174430	0.555046
H	-2.492065	2.223554	2.438503
H	-3.036401	4.019060	-1.426158
H	-3.949903	3.422057	0.815058
C	2.974046	-0.398169	-0.019357
C	3.917746	0.531590	0.450470
C	3.453803	-1.655308	-0.447267
C	5.272876	0.215313	0.497744
H	3.565142	1.502711	0.772468
C	4.808695	-1.965352	-0.401717
H	2.742419	-2.383578	-0.819549
C	5.731895	-1.031650	0.072652
H	5.977352	0.952684	0.870714
H	5.146042	-2.939389	-0.743386
H	6.789849	-1.271091	0.106371
S	-0.280625	-2.284352	0.220502
C	-2.057566	-2.058991	0.026079
C	-2.865537	-3.189662	0.200052
C	-2.640960	-0.834701	-0.301247
C	-4.245990	-3.093544	0.045925
H	-2.411022	-4.142382	0.456216
C	-4.023300	-0.747274	-0.454036
H	-2.005268	0.034576	-0.423223
C	-4.833890	-1.870654	-0.283827
H	-4.863576	-3.975808	0.184237
H	-4.464214	0.211954	-0.703548
H	-5.909608	-1.794916	-0.403196

TS1_@C4

C	0.732591	-0.240095	0.617830
C	1.965520	-0.489441	0.778846
S	0.141942	1.011306	-1.170770
C	-1.266272	1.966977	-0.689169
C	-1.124443	3.138910	0.080395
C	-2.570548	1.593030	-1.068093
C	-2.230689	3.899242	0.448667
H	-0.128263	3.440317	0.383601
C	-3.675545	2.357277	-0.702650
H	-2.700848	0.688732	-1.650934
C	-3.516527	3.516641	0.059470
H	-2.089226	4.796973	1.043834
H	-4.668633	2.042760	-1.010611
H	-4.378634	4.109725	0.347150
C	3.295662	-0.290775	0.363256
C	3.956804	-1.212119	-0.492567
C	4.060431	0.812247	0.829758
C	5.282751	-1.030545	-0.859983
H	3.403647	-2.068910	-0.860873
C	5.387171	0.977094	0.457687
H	3.584183	1.534577	1.482793
C	6.019598	0.061789	-0.390376
H	5.751084	-1.752101	-1.523708
H	5.937111	1.837531	0.828847
H	7.056716	0.196258	-0.677600
S	-0.688623	-0.568753	1.594397
C	-1.635208	-1.746749	0.651069
C	-1.165710	-2.364124	-0.513343
C	-2.905174	-2.086520	1.141644
C	-1.956713	-3.305724	-1.168240
H	-0.194323	-2.090794	-0.902812
C	-3.683580	-3.034602	0.485103
H	-3.282319	-1.599984	2.035407
C	-3.215543	-3.651486	-0.676739
H	-1.582168	-3.770992	-2.074603
H	-4.663590	-3.285697	0.878819
H	-3.825297	-4.385396	-1.192747

2_Z@C3

C	0.801219	0.653991	-0.327974
C	-0.521120	0.697391	-0.620955
S	-1.397448	-0.646156	-1.559930
C	-2.133295	-1.677605	-0.304386
C	-1.914372	-1.495549	1.067628
C	-2.961291	-2.734311	-0.719324
C	-2.514504	-2.345242	1.994029
H	-1.266792	-0.690520	1.394256
C	-3.551610	-3.582603	0.212651
H	-3.140496	-2.885569	-1.779004
C	-3.335694	-3.394042	1.579090
H	-2.332229	-2.185864	3.052366
H	-4.186799	-4.393536	-0.131230
H	-3.798603	-4.052953	2.305972
C	-1.356251	1.874353	-0.271790
C	-2.745754	1.940814	-0.492617
C	-0.759482	3.008200	0.326869
C	-3.491838	3.065931	-0.140367
H	-3.245077	1.097958	-0.952760
C	-1.503179	4.126617	0.675636
H	0.307958	2.972816	0.503797
C	-2.882992	4.172010	0.446468
H	-4.561839	3.071048	-0.328503
H	-1.002285	4.976762	1.130974
H	-3.463659	5.047273	0.719910
S	1.720310	-0.748997	-0.777082
C	3.372679	-0.381814	-0.187327
C	4.367224	-1.344142	-0.416048
C	3.713977	0.798641	0.481896
C	5.673591	-1.129886	0.015801
H	4.109649	-2.262821	-0.934755
C	5.022353	1.006457	0.910990
H	2.926838	1.526172	0.645426
C	6.012940	0.048268	0.683907
H	6.429664	-1.886957	-0.170307
H	5.271674	1.927882	1.429736
H	7.030599	0.215885	1.021253

2_E@C3

C	0.670045	0.194323	-0.669344
C	-0.611266	0.630241	-0.745626
S	-1.746241	-0.515387	-1.629118
C	-2.253258	-1.732674	-0.430659
C	-1.721154	-1.815344	0.863392
C	-3.226111	-2.670658	-0.818707
C	-2.164122	-2.800266	1.743815
H	-0.951508	-1.113479	1.159081
C	-3.657540	-3.655046	0.064701
H	-3.644816	-2.619435	-1.818985
C	-3.132741	-3.727155	1.357203
H	-1.739436	-2.845473	2.742141
H	-4.410827	-4.368068	-0.257309
H	-3.470647	-4.493019	2.047262
C	-1.269834	1.876773	-0.275588
C	-2.591949	1.895165	0.214613
C	-0.583394	3.107681	-0.287863
C	-3.180737	3.064480	0.685917
H	-3.161000	0.973959	0.220624
C	-1.175443	4.280089	0.175153
H	0.423941	3.144319	-0.682071
C	-2.478649	4.270994	0.671622
H	-4.197973	3.033135	1.065477
H	-0.614208	5.209293	0.139539
H	-2.939708	5.184066	1.034295
S	1.888336	1.150402	0.144373
C	3.335717	0.085893	0.085234
C	4.516645	0.560087	0.671953
C	3.331774	-1.175065	-0.517931
C	5.674226	-0.214920	0.655096
H	4.522749	1.539111	1.142277
C	4.492703	-1.943893	-0.532173
H	2.399795	-1.510133	-0.962439
C	5.671608	-1.474049	0.051980
H	6.581260	0.166504	1.114878
H	4.476926	-2.922383	-1.003904
H	6.572823	-2.078584	0.038321

2_@C4

C	0.006974	0.425931	0.549557
C	0.775543	1.377670	1.020005
S	0.263892	0.493868	1.059861
C	0.712666	2.143861	0.570207
C	1.159697	2.474950	0.716493
C	0.646490	3.160207	1.538463
C	1.528902	3.783216	1.020264
H	1.220290	1.700500	1.470910
C	1.030619	4.461949	1.231424
H	0.287863	2.921600	2.534145
C	1.472427	4.786366	0.052551
H	1.870063	4.017030	2.024112
H	0.974168	5.229306	1.997626
H	1.764419	5.802747	0.294299
C	2.006328	1.876381	0.501124
C	2.052721	3.006642	0.358250
C	3.258575	1.330654	0.891055
C	3.258121	3.534039	0.802099
H	1.118625	3.465211	0.665803
C	4.457467	1.867464	0.440461
H	3.268794	0.473393	1.555737
C	4.480232	2.977171	0.410627
H	3.245610	4.394434	1.466394
H	5.391985	1.410547	0.755881
H	5.419462	3.395305	0.756419
S	1.484476	0.134487	1.444154
C	2.860276	0.522349	0.526594
C	2.736606	1.534384	0.434133
C	4.140297	0.022706	0.818646
C	3.865728	2.028306	1.083544
H	1.755331	1.933193	0.657182
C	5.264199	0.530361	0.174702
H	4.247488	0.770831	1.551130
C	5.136520	1.536003	0.785767
H	3.748362	2.810854	1.826924
H	6.244077	0.129847	0.416391
H	6.011819	1.925827	1.294278
