# Supplementary material for: All-organic polymeric materials with high refractive index and excellent transparency
Source: Nat Commun. 2023 Jun 14;14:3524. doi: 10.1038/s41467-023-39125-w (PMC10267154; doi:10.1038/s41467-023-39125-w)
Supplement: Supplementary file 3 — Description of Additional Supplementary Files [file 41467_2023_39125_MOESM3_ESM.pdf]

Description of Additional Supplementary File

File Name: Supplementary Data 1
Description: Crystal structure of compound 6 (CCDC: 1889353) cultured in dichloromethane/n-hexane (1:1, v/v).
